# Commentary: Rheumatoid Cachexia Revisited: A Metabolic Co-morbidity in Rheumatoid Arthritis

**DOI:** 10.3389/fnut.2015.00037

**Published:** 2015-12-16

**Authors:** Thomas J. Wilkinson

**Affiliations:** ^1^School of Sport, Health and Exercise Sciences, Bangor University, Bangor, UK

**Keywords:** rheumatoid cachexia, cachexia, sarcopenia, inflammation, rheumatoid arthritis

The “mini review” by Masuko (1) contains a quite extensive summary of “rheumatoid cachexia” (2) [i.e., loss of muscle mass in rheumatoid arthritis (RA)], an often overlooked and misdiagnosed co-morbidity. Masuko looks at the definition of “rheumatoid cachexia,” as well as the pathogenesis of the condition referring to the key role of pro-inflammatory cytokines. Interestingly, various strategic therapy options designed to attenuate rheumatoid cachexia by disease-modifying anti-rheumatic drugs (DMARDS), anti-cytokine therapies, and exercise training (i.e., resistance and endurance) are covered.

However, what the review fails to mention or expand upon, is the relative ineffectiveness of exercise on “rheumatoid cachexia” once supervision is withdraw. The review also neglects to discuss (despite referencing a relevant trial) another potential key tool in the armory against “rheumatoid cachexia”: nutritional supplementation. This commentary will confine itself to briefly recounting some of these.

As Masuko states that resistance training is the most beneficial means to increase both lean mass and physical function in RA. Both Marcora et al. (3) and Lemmey et al. (4) showed that progressive resistance training (PRT) for a period of 12 and 24 weeks (exercising two and three times per week, respectively) was able to attenuate the effects of rheumatoid cachexia, specifically increasing lean mass, strength, and physical function in patients with RA. However, a follow-up study for 3 years later by Lemmey et al. (5) found that once supervision was withdrawn, no subjects from the original study (4) were performing relevant PRT or any other form of regular high intense exercise and, as a result, lean mass had regressed to baseline levels, as had much of the trunk fat mass and body fat percentage. Lack of adherence to high-intense exercise in RA has been demonstrated by others (6).

Unfortunately, it seems habitually performed, high-intensity exercise (i.e., PRT) is unlikely to be widely adopted as a general treatment strategy for reversing “rheumatoid cachexia” and restoring function, then the challenge to develop a treatment option that is easily administered, inexpensive, and makes limited demands of the patient remains. Anabolic nutritional supplementation offers a potential treatment option that is easily administered, inexpensive, and makes limited demands of the patient.

Scientific evidence continues to suggest that nutrition should be part of routine care in those with muscle wasting disorders [for review, see Stamp et al. (7)]. Specifically, up to 75% of RA patients believe that food and nutrition play an important role in their symptom severity, with 50% of RA patients reportedly trying some form of dietary manipulation in an attempt to attenuate symptomology (7). Marcora et al. (8) previously investigated the effects of daily 12 weeks of a mixture of β-hydroxy-β-methylbutyrate, glutamine, and arginine (HMB/GLN/ARG) protein supplementation in 40 RA patients. The results showed that both HMB/GLN/ARG and a control mixture of other non-essential amino acids (alanine, glutamic acid, glycine, and serine) were both equally effective in increasing lean mass (~0.4 kg) and improving some measures of physical function and strength.

Creatine monohydrate, a combination of essential amino acids, has generally been shown to be more effective in increasing lean mass and physical performance than other protein-based supplements, including HMB/GLN/ARG [for review, see Nissen (9)]. In the only study investigating the use of creatine in RA, Willer et al. (10) found that creatine supplementation increased muscle strength in 8 out of the 12 patients by an average of 14% as determined by the muscle strength index, although this increase in strength was not actually associated with changes of creatine (or phosphocreatine) levels in the muscle.

Nonetheless, creatine has been shown to be effective, without additional exercise training, in range of clinical populations, including muscular dystrophy patients and the elderly who, like RA patients, present with reduced muscle mass and impaired physical function [for review, see Wilkinson et al. (11)]. Evidently, more research is needed, and as such we have just completed a randomized control trial investigating the effects of creatine in RA patients (manuscript in preparation).

To summarize, although PRT exercise is arguably the most effective way to attenuate the effects of rheumatoid cachexia, due to its high-intense nature, it is unlikely to be widely adopted as a general treatment strategy. Nutritional supplementation, specifically those that focus on muscle anabolism may be a viable option. Alongside, HMB/GLN/ARG protein supplementation, creatine monohydrate may be a potential therapeutic option in RA, as it has been effective in range of clinical and elderly populations.

## Conflict of Interest Statement

The author declares that the research was conducted in the absence of any commercial or financial relationships that could be construed as a potential conflict of interest.
